# Lived Experiences of Female Nurses with COVID-19 Deaths on Their Watch

**DOI:** 10.3390/bs12120470

**Published:** 2022-11-23

**Authors:** Mai B. Alwesmi, Wireen Leila Dator, Savvato Karavasileiadou

**Affiliations:** 1 Department of Medical-Surgical Nursing, College of Nursing, Princess Nourah bint Abdulrahman University, P.O. Box 84428, Riyadh 11671, Saudi Arabia; 2 Department of Community Health Nursing, College of Nursing, Princess Nourah bint Abdulrahman University, P.O. Box 84428, Riyadh 11671, Saudi Arabia

**Keywords:** COVID-19, death, nurse

## Abstract

The COVID-19 pandemic has had a tremendous effect on health systems, leading to a spike in stress, anxiety, and depression conditions among healthcare workers worldwide. Considering the mental health status of nurses, a pillar of the health system, is crucial for assuring the quality of the healthcare provided during and after pandemics. This study aimed to explore the experiences of female nurses who witnessed their patients dying of COVID-19. Seven female COVID-19 nurses were interviewed in person. Thematic analysis of the respondents’ verbatim answers was implemented. Six main themes were identified. Theme 1 illustrates nurses’ experience of emotional and psychological trauma as they witnessed their patients with COVID-19 die under their care. Theme 2 reveals aftershock reactions, including somatization, isolation, and emotional disengagement. Theme 3 highlights the hindrances and difficult situations related to the in-hospital care of COVID-19 patients. Theme 4 demonstrates victorious feelings and celebration of the patient’s survival. Theme 5 emphasizes the nurse’s ability to be calm and recognize the takeaways or lessons they have learned from this phase for their careers and lives. Theme 6 sheds light on nurses’ emerging abilities and competencies. This study will hopefully provide a basis for a debriefing program that might be beneficial to the nurses and the health system. This might affect nurses’ ability to work closely with patients, cope emotionally with challenges, and fulfil their professional tasks.

## 1. Introduction

The coronavirus disease 19 (COVID-19) pandemic has had a tremendous impact on health systems, leading to a spike in stress and anxiety conditions amongst populations worldwide, including amongst healthcare providers. Coronavirus disease 19 (COVID-19) is a novel respiratory viral disease that ranges from mild-to-moderate illness to severe progressive pneumonia, multiorgan damage, and death. It threatens individuals of all ages, genders, and races. The first case of this disease was reported on 31 December 2019 in Wuhan, the capital of Hubei Province in the People’s Republic of China. It promptly reached 20 countries around the world. On 30 January 2020, the World Health Organization (WHO) declared the outbreak a global health emergency. Thereafter, on 11 March 2020, the WHO declared COVID-19 a pandemic.

The worldwide mortality toll rose dramatically in a relatively short period of time. As of 15 November 2022, there has been over 632,533,408 cases and 6,592,320 deaths worldwide since 21 December 2019 (WHO COVID-19 Dashboard, 2022). These figures have had a tremendous impact on health systems, leading to a spike in stress and anxiety conditions amongst populations worldwide, including amongst healthcare providers. As nurses are at the frontline of the healthcare workforce, a higher rate of stress was expected to be found among them. In Saudi Arabia, over 815,839 cases and 9342 deaths have been recorded, which is considered one of the lowest rates in the world (COVID-9 Dashboard: Saudi Arabia, 2022; WHO COVID-19 Dashboard, 2022). However, considering the effects of COVID-19 on nurses is deemed necessary in view of recent findings by AlAteeq et al. (2020), who estimated the prevalence of depression and anxiety among healthcare providers in Saudi Arabia during the COVID-19 pandemic [1]. This study showed that female practitioners, including nurses, presented higher levels of anxiety compared to male ones.

Conversations and discussions with female nurses at King Abdullah bin Abdulaziz University Hospital (KAAUH) shed light on the mental health issues nurses might have developed after witnessing the deterioration and death of COVID-19 patients. They expressed a sense of helplessness due to the challenges of providing adequate patient care and the fear of infection with insufficient control procedures, in addition to the uncertainty of workplace response to the pandemic. Published data on outbreaks of previous pandemics such as the Ebola virus disease in West Africa in 2014–15 [2] and severe acute respiratory syndrome (SARS) in Taiwan in 2003 emphasized the catastrophic impact of viral infectious diseases pandemics on mental health [3]. Likewise, the COVID-19 pandemic has had a tremendous effect on health systems, leading to a spike in stress, anxiety, and depression conditions among populations worldwide [4,5,6] and among healthcare workers [7,8].

Stress, anxiety, and depression are common mental health problems that may interfere with daily functioning. According to the WHO, mental health is defined as “a state of well-being in which an individual realizes his or her own abilities, can cope with the normal stresses of life, can work productively, and is able to make a contribution to his or her community” (WHO, 2021). Portoghese et al. (2021) highlighted the need for psychotherapeutic interventions for health practitioners who dealt with COVID-19 patients during the first two months of the pandemic in Italy [9]. Several editorials, by Jackson et al. (2020), Maben (2020), and Smith (2020), highlighted nurses’ need for psychological support while dealing with the challenges of COVID-19 outbreaks. These editorials outlined mental health outcomes observed among nurses worldwide during the pandemic. Nurses are at the frontline of the healthcare workforce, working daily for long hours at the bedside, dealing with patient deterioration, and providing end-of-life care; therefore, they were expected to be mentally exhausted. These editorials also pointed out the lack of psychological support for nurses while working under pressure, surrounded by stressors and challenges [10,11,12]. There is mounting evidence that nurses experience significant psychological strain as a result of witnessing patients’ suffering, particularly the terrible end of a patient’s life [13,14].

In the past 19 years, there have been several viral epidemics; in 2003, severe acute respiratory syndrome (SARS) occurred; in 2009, the virus subtype H1N1 caused influenza; a Middle East respiratory syndrome (MERS) epidemic occurred in 2012; and, in 2014, a Ebola virus disease epidemic broke out [15]. These outbreaks raised problems for healthcare workers in terms of the psychological effects of pandemics such as stress, depression, and post-traumatic stress disorder (PTSD). However, nurses were at a higher risk of adverse psychological experiences than doctors or other healthcare workers [15]. These issues were especially acute during the COVID-19 pandemic [16]. Nurses faced challenges related to having direct contact with COVID-19 patients, lack of protective equipment, social isolation, danger and fears of infection, and increased workload [17]. Based on the results of 36 studies on the mental health status of nurses during the COVID-19 pandemic, Mahalli and Nematifard (2021) concluded that the following negative psychological effects were observed: stress, depression, anxiety, sleep disorders, fear in the medical staff, and PTSD. Some studies showed burnout, mistrust, irritability, hopelessness, and suicidal ideation [18]. The above negative psychological experiences were most pronounced among nurses on the frontline.

Researchers noted that the scenes witnessed by nurses caused various adverse psychological experiences, namely a sense of helplessness [19], frustration [19,20], fear [6], depression [21,22], anxiety, and stress [22]. Younger nurses (aged less than 40 years old) had a relatively higher stress level [23]. Women were more susceptible to stress than men [24]. A lower educational level of nurses was associated with a lower level of mental distress and depression [25]. Arnetz et al. (2020) noted that some of the strongest sources of stress were witnessing the deterioration of patients, witnessing their death, and caring for extremely ill or ventilated patients [20]. Nurses reported that they were sad and stressed due to the mortality of the pandemic [6]. One of the interviewed nurses described her impressions of her patients’ deaths as follows: “When a patient with an infectious disease dies, the body is wrapped in several layers of cloth, packed into two bags, which are sprayed with disinfectant. It was a little hard to accept this form of death” [21].

To estimate the prevalence of mental health problems in nurses during the COVID-19 pandemic compared with the prevalence of these problems during the MERS and SARS epidemics, Al Maqbali et al. (2021) conducted a meta-analysis. This meta-analysis was based on 93 cross-sectional studies in which 93,112 nurses participated [26]. The results showed high proportions of mental health problems, namely the prevalence of anxiety, stress, depression, and sleep disturbance was 37%, 43%, 35%, and 43%, respectively. The authors concluded that at least one-third of nurses had anxiety, stress, depression, and sleep disturbance. The prevalence of these symptoms was higher than in the general population and higher than during the MERS and SARS epidemics [26]. This figure can be explained by the fact that the virus is transmitted from person to person, spreads rapidly, and is characterized by high mortality.

Considering the mental health status of nurses, a pillar of the health system, is crucial for assuring the quality of the healthcare that is provided after the pandemic. Their well-being status determines their ability to work closely with patients, cope emotionally with challenges, perform social responsibilities, and fulfil their professional tasks. Alterations in their mental health closely relate to physical health, leading to improper functioning in work activities and putting patients and care providers at risk [27]. Determining the factors of a mental health condition facilitates assessment and diagnosis, clinical management, therapeutic interventions, and complication avoidance.

## 2. Materials and Methods

### 2.1. Procedure

This study employed a descriptive, qualitative design to capture the unique experience of nurses whose patients were diagnosed with and died of COVID-19 during their watch. It looked into the impact of the experience on their well-being. Interviews were conducted at King Abdullah bin Abdulaziz University Hospital, Riyadh, Saudi Arabia. An interview protocol was developed prior to the data gathering. The protocol included several phases and activities that included: preparation of the semi-structured questions and piloting among the researchers and two other nurses; establishing rapport between the interviewer and the participants prior to the interview; securing consent; establishing duration, time, and venue for the interview; ensuring the safety and comfort of both the interviewer and the participants; and the documenting and storage of the recorded interviews and fieldnote transcripts (detailed interview protocol is available upon request). The grand tour question asked was: “Can you tell me your experience witnessing your patient diagnosed with COVID-19 die during your duty?” Subsequent questions were asked based on their answers. The gathered information was recorded with their consent. Themes were extracted from the narratives.

Prior to the interview and during the analysis, reflexive bracketing was performed to avoid subjective bias during questioning and in the analysis of the information. Note-taking and recording with the participant’s consent were carried out. The feelings and expressions of the participants during the interview were documented in order to add meaning to the words expressed.

### 2.2. Sampling and Sample Size

Participants in this study were purposively selected based on the set inclusion criteria. The participants had to be nurses who took care of patients diagnosed with COVID-19 and witnessed one of their patients dying; were able to speak and understand the English language; and consented to participate in the study. Data saturation was used to determine the sample size. Saturation is accepted and used in qualitative research as a criterion to stop the data collection and/or analysis. Saturation as a basis to stop sampling the group is when there are no additional data coming out with which the researcher can develop properties of the category. As he sees similar instances over and over again, the researcher empirically secures that a category is saturated. In this study, data saturation was reached after seven interviews; hence, the sample size was seven.

### 2.3. Data Analysis

Qualitative data analysis of the verbatim answers of the respondents, and thematic analysis in particular, was conducted. A secondary reviewer from the university, in addition to one of the co-authors, was involved to ensure that interpretation and analysis of the verbalizations and emerging themes were correct. Colaizzi’s (1978) approach was employed following the seven steps: familiarization with the data gathered by reading the transcripts several times, followed by identification of significant statements that are relevant to the phenomenon being investigated; formulating meanings from the significant statements considering reflexive bracketing; clustering of the formulated meanings into themes; writing exhaustive descriptions that capture the structure of the phenomenon; and the final stage where the respondents are asked to confirm that the correct themes have been captured. Necessary modifications may be performed based on the feedback of the participants [28].

## 3. Results

### 3.1. Profile

Seven female nurses were interviewed. Their ages were 25, 31, 33, 38, 44, 47, and 58 years old; five of them were married, and the other two were single. Their nationalities included Indian, Saudi, Filipino, and African. Three of them had less than 10 years of work experience as a nurse and the rest over 15 years. Four of them were Muslims, and three were Christians. All of them worked more than 12 hours per day with a maximum of a week of not going home for those living outside the hospital.

### 3.2. Emerging Themes

**Figure 1:**
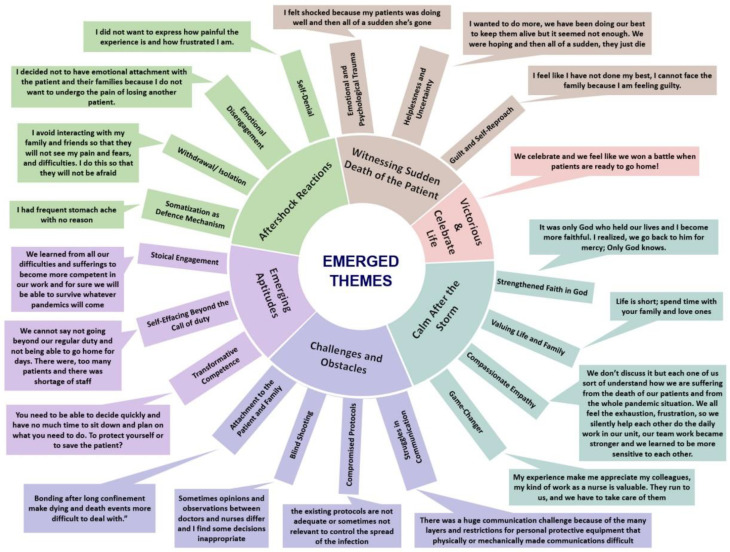
A schematic illustration of emerged themes and subthemes with relevant participants’ quotes.

Analyses of the obtained data from the female nurses who witnessed their patients dying of COVID-19 resulted in six themes, as summarized in Table 1 and Figure 1. Theme 1: Witnessing Sudden Death of the Patient with subthemes Emotional and Psychological Trauma, Helplessness and Uncertainty, Guilt and Self-Reproach; Theme 2: Aftershock Reactions with subthemes of Somatization as a Defense Mechanism, Withdrawal/Isolation, Emotional Disengagement, and Self-Denial; Theme 3: Challenges and Obstacles with subthemes of Struggles in Communication, Compromised Protocols, Blind Shooting, and Attachment to the Patient and Family; Theme 4 with no subthemes: Victorious and Celebrate Life; Theme 5: Calm After the Storm with subthemes of Strengthened Faith in God, Valuing Life and Family, Compassionate Empathy, and Game Changer; and the last theme: Emerging Aptitude with the subthemes Transformative Competence, Self-Effacing Beyond the Call of Duty, and Stoical Engagement.

#### 3.2.1. Witnessing Sudden Death

This theme captures the emotions and psychological state of the nurses who witnessed patients dying suddenly or unexpectedly after a long time of confinement and an attachment had been developed between the nurse and the patient. The subthemes provide experiences that are more specific.

##### Emotional and Psychological Trauma

Emotional and psychological trauma is a consequence of exposure to unexpected or highly stressful life events that cause a loss of psychological security and stability, leaving the individual with feelings of excessive fear and helplessness [29]. Subjects under psychological trauma might feel emotionally numb, experience a sense of disconnection, and lose trust in others. Psychological trauma is usually associated with life-threatening situations, though an individual’s responses to anxiety triggers and depression stimuli might lead to trauma [29]. The nurses experienced emotional and psychological trauma as they witnessed their patients with COVID-19 die under their care. They identified an array of emotions that they felt during these deaths and dying events. These emotions included: shock, sorrow, regret, guilt, disbelief, frustration, crying, emotional burden, and betrayal.

*N1. “I felt shocked because my patient was doing well and then all of a sudden she’s gone. The long stay of the patient had developed our attachment and her progress made us hopeful that she would make it. It is a very sad event.”*

*N2. “It is always a very sorrowful time when our patients go all of a sudden. This virus betrayed us. Our patients were showing some progress and then all of a sudden they just give in and die. I cannot help but cry. I am shocked.”*

*N3. “They were always hopeful when I talk to them and now I cannot even mourn with them. I am in disbelief; I am so sad.”*

*N4. “After all that you have done, trying your best to keep the patient alive, I just can’t believe that they go all of a sudden. It is painful, shocking.”*

*N5. “I carry on my shoulder the heartbreak of the family. We feel frustrated and betrayed by this virus. I cannot believe my patient is gone after all that we have done.”*

*N3. “I cannot believe that my patient died all of a sudden, she was just coping well a moment ago.”*

##### Helplessness and Uncertainty

The nurses suffered from an overwhelming feeling of helplessness that was heightened by uncertainty upon the death of their patients.

*N2. “I cannot do anything.”*

*N3. “I have done everything I can, but nothing worked.”*

*N5. “We worked altogether but it was not enough.”*

*N6. “We do not know what was going on and what was going to happen next.”*

*N7. “The virus is different from the other experience and less is known about it.”*

*N6. “I wanted to do more, we have been doing our best to keep them alive but it seemed not enough. We were hoping and then all of a sudden, they just die.”*

##### Guilt and Self-Reproach

The nurses suffered guilt and self-reproach from the death of their patients. Guilt is a feeling experienced by individuals in the wake of their misdoing [30]. Self-reproach is harsh blame of oneself for misdoing and imagined transgressions or a sense of inadequacy [30]. While guilt might lead to constructive behavior change, self-reproach might be counterproductive [30]. Specific feelings deduced from their verbalizations included regrets for not being able to save the patient, self-blame, and anger towards themselves and another health team.

*P1. “The long stay of the patient had developed our attachment and her progress made us hopeful that she will make it. I felt I could have more.”*

*P2. “I can’t help but cry. It is all I can do. I should have done more.”*

*P3. “I feel like I have not done my best, I cannot face the family because I am feeling guilty.”*

*P4. “I could have done more.”*

*P5. “I regret that I was not able to save her.”*

*P6. “I feel angry with some of the health team, they were not aggressive to save the patient.”*

*P5. “The death happens so suddenly, and it made me feel so frustrated and guilty for not being able to save her.”*

#### 3.2.2. Aftershock Reactions

The nurses not only endured the impact of witnessing patients dying, but all of them bore extended effects even after the event. The subthemes from the verbalizations of the nurses illuminate the aftershock reactions.

##### Somatization As a Defense Mechanism

According to the unconscious theory, somatization is a strategy for an individual to avoid experiencing too much emotion. Psychodynamic theory views somatization as an ego defence, the unconscious rechannelling of repressed emotions into physical symptoms as a symbolic form of communication [31]. The majority of participants exhibited varied patterns of physical symptoms including stomach-ache, menstrual delays, and headaches and became easily irritated with simple or small things:

*N1. “I had a frequent stomach ache for no reason.”*

*N3. “Feeling tired for an unknown reason.”*

*N4. “I used to have regular menstruation, and now I have menstrual delays.”*

*N6. “I feel physically weak and lost interest in so many activities I used to be doing.”*

##### Withdrawal/Isolation

Withdrawal is a complex divergence from typical behavior influenced by factors such as age, culture, economic status, etc. It is considered a coping method that does not necessitate delusions of reality [32]. Participants described multiple feelings/mixed emotions of sadness, anger, and frustration that could not be dealt with or confronted and lost the desire to interact and engage in social activities:

*N2. “I go straight to my room to avoid my family and not to show them my frustrations and fears.”*

*N3. “I tell my family that I am okay and that they should not worry about me at all because I don’t want them to worry about me. But deep inside me, I feel angry, afraid, I have so many negative emotions.”*

*N5. “We just go on with our work and deal with our colleagues without talking about our fears and emotions but we all know how each one of us feels.”*

*N6. “I avoid interacting with my family and friends so that they will not see my pain and fears, and difficulties. I do this so that they will not be afraid.”*

##### Emotional Disengagement

Emotional disengagement is a maladaptive coping strategy that people use to cope with stressful situations [33]; it leads to adverse consequences [34]. Participants expressed a desire for keeping professional relationships and having less emotional bonding with patients and their family.

*N1. “Only the kind of relationship will change but not the dedication and commitment to patient care.”*

*N2. “I avoided to mingle with my family for fear to infect family, for family to have the infection and possible death.”*

*N1. “I decided not to have emotional attachment with the patient and their families because I do not want to undergo the pain of losing another patient. But I will continue to provide quality and caring service.”*

*N7. “You feel how the other nurses are also in pain and have the same feeling as you do, but you try to set aside this emotions and not confront or face it because there is so much work to do for the patient and also you do not have the strength to confront these emotions, so you suppress them at the moment.”*

*N3. “So you have to deal with so many deaths, and there is no time to grieve because there is so much to do.”*

*N4. “You feel so frustrated, sad, and even angry but you cannot show the family how vulnerable you are.”*

*N5. “You have to be the source of strength for the family of the patient and cannot show them you are weak because it will break them.”*

*N6. “It is so difficult to deal with the loss because of physical restrictions. And this is more painful when you cannot even touch the person you cared for… and how much more painful is this to the family.”*

##### Self-Denial

Denial, suppression, or the concealment of negative feelings are maladaptive coping techniques for distressing circumstances [29,35]. The nurses opted for repression of pain and painful experience more often than expressing their emotions.

*N1. “I did not want to express how painful the experience is and how frustrated I am.”*

*N2. “Avoiding dealing with feelings.”*

*N3. “Comforting oneself by distraction from the thought of the event.”*

*N4. “Setting aside feeling and tending to the patient and family, and other duty.”*

*N7. “I sleep to comfort myself at least for a while.”*

#### 3.2.3. Challenges and Obstacles

Challenges and obstacles refer to the hindrances and difficult situations that confronted the healthcare in the hospitals when taking care of COVID-19 patients, which included self-protection protocols that were not updated; too many patients, the number of which was continuously increasing at a very fast pace; communication challenges; shortage of staff and resources; inappropriate decision making and coordination among health teams; and dealing with too many deaths.

##### Struggles in Communication

*N3. “There was a huge communication challenge because of the many layers and restrictions for PPEs that physically or mechanically made communications difficult.”*

*N2. “The turn of events is too fast and there were too many PPE procedures and protocols that delay actions or slow down the delivery of services.”*

##### Compromised Protocols

*N1. ”There was no clear and updated self-protection protocols to follow since the virus is new and has a different mechanism compared with the previous breakouts we have had.”*

*N1, N2, N7. “Little is known about the virus, so the existing protocols are not adequate or sometimes not relevant to control the spread of the infection.”*

##### Blind Shooting

*N4. “Sometimes opinions and observations between doctors and nurses differ and I find some decisions inappropriate.”*

*N5. “Lack of experience in handling new cases with so many cases at the same time is so risky.”*

*N6. “The real situation is much more different from the simulated cases during training.”*

##### Attachment to the Patient and Family

One of the things that made the situation more emotional was the attachment between the nurse and the patient; being together on the journey from admission to staying in the hospital for a very long period developed ties.

*N1. “I feel so helpless, cannot do anything about the fate of the patient. I feel so sorry for the family who was hoping to bring home their loved one.”*

*N3, N4. “Bonding after long confinement makes dying and death events more difficult to deal with.”*

*N5. “We feel the void, after all, that you have tried to do, they became a family to us.”*

*N6. “Disappointed and devastated- after hoping and giving the best care for a long period of time and the patient dies.”*

*N7. “Exasperated from quick change from responsive to sudden death. It is heart-breaking to see the family who has become your family, too.”*

##### 3.2.4. Victorious and Celebrate Life

Every survivor among the patients sparked hope among the nurses and inspired them to believe that there was an opportunity to outlive the virus and its harmful effects.

*N1. “Everyone is Ecstatic for patient recovery after a very long period of confinement in the hospital and isolation from everyone.”*

*N2, N3, N5, N6. “Relieved when the patient is managed and survived.”*

*N1, N2, N3, N4, N5, N6, N7: “We celebrate and we fill like we won a battle when patients are ready to go home!”*

##### 3.2.5. Calm after the Storm

The experience of the nurse during the pandemic was generally horrible. Although the unpleasant experience lingers in their memories, they were able to regain being calm and recognize the takeaways or lessons they learned from this phase in their careers and lives. All of the nurses dearly held onto the following from their experience:

##### Strengthened Faith in God

*N1. “It was only God who held our lives and I become more faithful. I realized, we go back to him for mercy; Only God knows.”*

*N2. “I become closer to God and I found myself looking at how I took for granted my faith.”*

*N3. “It’s only God who knows what will happen to us.”*

*N4, N6. “I become more faithful and held on to God.”*

##### Valuing Life and Family

The participants realized that the everyday things and people that they took for granted could so easily be valued instead of emphasizing bad events or inadequacies. They learned how to practice gratitude as the appropriate response to beneficence by focusing on their families and whatever simple things they had rather than material things to nurture and sustain life and mental balance. Gratitude is a complex phenomenon referring to the appreciation of everything that you have in life. These positive emotions simply helped them in overcoming adversity and building their resilience [36].

*N2. “Life is short; spend time with your family and loved ones.”*

*N4, N5, N6. “Take care of love ones, you never know what happens.”*

*N7. ”Unpredictable things happen in life, don’t take your family for granted.”*

*N5. “Be the source of relief and reassurance for the family.”*

##### Compassionate Empathy

This is the emotion that motivates us to help others by going above and beyond merely understanding and experiencing their sentiments.

*N1, N3, N7. “We don’t discuss it but each one of us sort of understands how we are suffering from the death of our patients and from the whole pandemic situation. We all feel the exhaustion and frustration, so we silently help each other do the daily work in our unit, our teamwork became stronger and we learned to be more sensitive to each other.”*

*N1, N2, N6. ”We became the source of strength for the families who were not able to see their sick loved ones. So, we see to it that they are updated, made possibilities for them to see and communicate visually with their loved one.”*

*N2, N3, N5. “We cannot help but cry with the family sometimes.”*

##### Game Changer

This refers to a drastic alteration in normative behavior or way of thinking. The varied experience, from the care and management of the patient and their families to how they worked within their units and with other health teams, led them to realizations that were mostly geared towards gratefulness and appreciation of what they had around them more than towards complaining of the setbacks.

*N1. ”I was so upset from the inequality or overlooking of nurses’ needs—no debriefing, no psychological intervention available for us. This made me realize how we are undervalued, I am not sure if it is because the focus is on the patients. We have to learn how to survive on our own not depending on others, not even our managers.”*

*N5. “You will see the impartiality- some have hazard pay others none, and there is no recognition of efforts and risks. It hurts but I have to look beyond this and be rewarded from above with whatever you have done.”*

*N4. “The pandemic changed the way I looked at life. Show your love to your family now and all the time. Life is short and we never know what comes next.”*

*N2. “Focus on more essential things in life, no amount of material things, power, or influence, can spare you from the pandemic.”*

*N3. “Our working relationship changed. We have better Teamwork, we help each other more, and the pressures and stress we have from the pandemic made us bond more.”*

*N6. “My experience makes me appreciate my colleagues, my kind of work as a nurse is valuable. They run to us, and we have to take care of them.”*

#### 3.2.6. Emerging Aptitude

It is during difficult times that one’s capacity is put to the test. The nurses not only learned lessons in life, but their inner capabilities also emerged.

##### Transformative Competence

Inadequate preparation and work pressure developed their ability to adapt to ambiguity, grow innovative ideas and ideals, and continue to operate in a productive and meaningful manner in the face of altering goals.

*N1. “You have to learn how to manage the situation while there is no existing guideline set.”*

*N2. “The simulated training was not adequate to equip you on how to deal with so many cases you are unfamiliar with; so you need to deal with the situation based on the need and resources you have against restrictions.”*

*N3. “You learn how to be more assertive in terms of communication and leadership skills especially when some team members are indecisive.”*

*N4. “You need to be able to decide quickly and do have no much time to sit down and plan on what you need to do. To protect yourself or to save the patient?”*

*P5. “You become resourceful when there are not enough resources available.”*

*N6, N7. “Whatever you have learned in your education and training is not enough, during unfamiliar and unpredictable situations, you have to learn how to handle your emotions, be able to think clearly, set aside differences, focus on your goal, and most of all learn how to be independent, assertive, resourceful and decisive.”*

##### Self-Effacing beyond the Call of Duty

Self-efficacy is a belief of an individual in his or her ability to complete a particular task and exert control over his or her motivation, behavior, and environment [37]. During the pandemic, the nurses, despite their fears and the fact that they were exposed to higher risks of contacting the dreadful COVID-19, experienced a sense of responsibility that extended beyond the self. They continued to provide their services as frontlines beyond what was legally required from them, services that went beyond the norm or standard to keep the patient alive.

*N1. “We cannot say no going beyond our regular duty and not being able to go home for days. There were too many patients and there was shortage of staff.”*

*N2. “Who will take care of them if we will not extend our services and duty on a 24/7 basis?”*

*N3. “I forgot about my family and myself even if I fear for them contacting the virus, I have to be in the hospital to perform my duty.”*

*N5. “We had no choice if we were rotated way more than the regular duty that we are legally bound to deliver, the cases were increasing exponentially.”*

*N6. ”Duty first before self.”*

*N7. “We don’t count, we just do our duty. No one else will do these if we do not. This is what we promised as professionals, and as humans, we help others as long as we can.”*

##### Stoical Engagement

This is being able to endure hardship without showing emotion or complaining [38].

*N1. “We have become more supportive of each other and our teamwork is much stronger.”*

*N2. “We learned from all our difficulties and sufferings to become more competent in our work and for sure we will be able to survive whatever pandemics will come.”*

*N4, N7. “This pandemic made us suffer a lot but it also turned us into more competent nurses. It made me realize how important my profession is and inspired me to do better.”*

## 4. Discussion

This study investigated the impact of witnessing patients’ deaths during stressful times on female nurses. Nurses reported that they experienced strong stress as a result of witnessing the suffering and death of patients [6,20,22,39,40,41,42] and the handling of the deceased [6,21].

Despite uncertainty over the clinical course of COVID-19, the urge to serve others was a strong motivator for the participants. However, research has shown that this drive is associated with increased burnout and other physical symptoms, such as poor self-perceptions and emotions [43]. The uncertainty over the clinical course of COVID-19, along with other personal and professional concerns, is a theme that has emerged from all interviews in recently published studies. The nurses described their experience during this time of uncertainty as surreal and unreal since they were unprepared for it [44]. Due to a relatively low rate of improvement in patients’ conditions and high mortality, nurses felt unable to provide the necessary care to their patients [20,39,42,45,46].

Nurses expressed concerns about the quality of care they provided [19,22,39,47,48]. In addition, they reported that they had to take care of patients while the operating procedure was not clear enough [22,45,47,48,49]. Nurses noted that the healthcare system was not prepared for the pandemic, and the guidelines were unclear, incomplete, and continuously evolving [6,49,50,51,52,53,54]. Since it was impossible to establish validated diagnostic protocols in the midst of a crisis such as COVID-19, nurses’ post-pandemic reflections might contribute to the efficient handling of future crises.

A sense of guilt stemmed from the carelessness caused by the tight care standards established; for example, by wearing PPE prior to contact with patients, they were unable to deliver full care [14]. The above flaws caused moral distress in nurses [6,30,46,48]. As a result, nurses may have experienced burnout, compassion fatigue, and a decline in their overall health and happiness [55]. To better deal with these challenges, nurses should seek out psychological counselling. During the pandemic, healthcare workers were honored in a way that emphasized their self-denial. Normalizing nurses’ risk exposure, enforcing model citizenship, celebrating heroic and self-sacrificing nurses, and preserving power connections are all examples of phenomena that limited frontline nurses’ ability to set the conditions of their job [32]. Institutions must adopt structural strategies that promote nurses’ self-care knowledge so that they can provide better care for others during and after a pandemic [56,57].

The lack of pandemic knowledge caused frustration and a sense of loneliness in nurses [58]. Quarantining and social distancing reduced mortality and morbidity but caused social isolation and stigmatization [4]. Social isolation and quarantine exacerbated nurses’ anxieties and harmed their performance and mental health [59]. Attempts have been undertaken to boost healthcare personnel’s morale. Despite these efforts, certain segments of society continue to stigmatize healthcare providers. According to some studies, society’s reactions made healthcare professionals feel guilty and desire a life of social seclusion and limited contact with the outside world [4,58]. Despite following isolation requirements, some nurses were labelled as disease carriers. They avoided their families, residences, and social environments out of fear of contagion [60]. Female nurses who took part in a previous study reported that they struggled to meet societal expectations such as caring for the elderly and raising children while keeping up with the ever-growing demands of personal hygiene [46]. Due to the lack of rules and guidelines, nurses had to adjust to new routines and deal with the emotional toll of being away from their loved ones. They were left to deal with their uneasiness and fears on their own. However, self-efficacy was among the main protective factors preventing negative psychological experiences during the COVID-19 pandemic in addition to psychological resilience and intra-family and extra-family social support [61,62].

As patients lay dying, nurses often filled the role of a surrogate family member. Nurses were troubled by the shift in policy, the exclusion of family members, and the segregation of dying patients [44]. Nurses reported that there was an insufficiency in the emotional and psychological support they could provide to patients and their families, and one of the main reasons for this was the lack of relevant knowledge [42,46,50]. Nurses were unable to regulate their emotions and avoid feeling the suffering of others. To develop protective self/other boundaries, they must be able to recognize the suffering of others, which is referred to as the self/other distinction. It requires firstly self-awareness and then self-management of emotions [35]. Self-awareness skills and emotional management are essential for maintaining overall well-being and regulating an individual’s capacity for compassion [56].

The stoical engagement phenomenon observed among the participants supports the findings of recently published case studies on resilience [63]. Psychological resilience is an individual’s ability to cope with stressors or traumas and to recover quickly from crises [64]. A female nurse described her experience in attending psychologist sessions for nurses during the first wave of COVID-19 [63]. She stated that nurses, from her perspective, have a tendency toward stoicism and a can-do attitude, which is related to their professional pride. Nurses have internalized the concept of resilience, which has had a detrimental effect on their mental health and kept them from receiving treatment when it was most necessary. The assumption that stoicism and resilience are interchangeable was widespread in a recently published study [63]. This might be due to stereotyped nursing feelings [65].

The psychological impact of the COVID-19 outbreak undoubtedly exacerbated self-reported somatic symptoms among frontline healthcare workers [21,66], particularly among female nurses [67]. Healthcare workers adopting emotional disengagement as a coping strategy demonstrated significant clinical anxiety compared to healthcare workers who adapted planning strategies [23]. A recent cross-sectional study showed that, in comparison to physicians, nurses experienced greater distress and burnout and employed more maladaptive coping mechanisms [68]. Some mental symptoms might be so severe that they prevent a person from thinking clearly. The individual’s stress may then manifest physically as a symptom, which, in turn, causes substantial physical and mental distress. This emphasizes the need for a deeper understanding of psychological distress and coping mechanisms among professionals giving care to patients throughout a pandemic. Future research should concentrate on the development of interventions that educate nurses on the application of particular coping techniques.

Being self-effacing beyond the call of duty added to the already growing workloads brought on by inconvenient COVID-19 protocols [69]. It was found that self-efficacy, psychological resilience, and social support were negatively associated with anxiety, depression, and PTSD [17,61]. They were also good protective factors against nurse burnout [61,62]. As found in a previous study, intensive care nurses and other nurses will likely collaborate more in the future. Working with new colleagues highlighted the importance of excellent communication, cooperation, and team leadership [70].

When patients made progress in their recoveries, there was cause for celebration, especially when a patient was finally able to leave after an extended period. This not only served as a treat for the nurses after their arduous journey but also as an example of the hope that was still very much alive. This phenomenon was consistent with a recent study where nurses demonstrated feelings of happiness when patients recovered, especially when they were discharged from the intensive care unit. They referred to this as a win over the sickness, and it gave the nurses hope for future patients and the will to continue their work. In addition, the nurses recounted numerous emotional occasions, such as when patients were able to connect with their relatives for the first time in perhaps weeks [70].

Nurses realized the necessity of professional solidarity. In a study by Muz and Yüce, 2021, nurses appreciated their colleagues and provided greater mental and social support for one another [46]. Among all possible resources, nurses relied on their colleagues the most when trying to achieve a healthy mental state. Their colleagues’ encouragement gave them the willpower to care for patients whose conditions were deteriorating or who were having life support removed [45]. Nurses reported growing in their professional identity. Recognition of the importance of nurses, the teams’ greater trust in their abilities, the nurses’ success in helping their patients recover, and the nurses’ own personal development emerged as a result of the crisis [40]. Nurses’ job satisfaction rose as a result of the widespread pandemic. The difficulties nurses had to face strengthened their determination and unveiled their actual potential [45].

The valuing of life and family observed among the participants supports four previously reported case studies of nurses who practiced gratitude toward the positive things in life that assist them in conquering obstacles and developing their resilience [71]. This phenomenon has also been observed among patients who have recovered from COVID-19 [72]. Established connections and the optimism they inspire take centre stage as we emerge from COVID-19 restrictions, i.e., enhanced bonds within social circles, including the development or maintenance of closer relationships with family and friends, and an increased willingness to aid others.

The majority of respondents demonstrated that the COVID-19 pandemic strengthened their faith in God. Faith is a strong booster for optimism and hope [73]. Faith in God and spiritual growth assisted nurses in maintaining their psychological health. It was one of the coping techniques adopted by nurses during the pandemic that lowered their stress level [74]. Recent studies have established a link between religious beliefs and practices and beneficial health outcomes such as illness resilience, hospital readmission rates, and mental wellness [75,76,77]. Alquwez et al. (2022) concluded that nurses’ faith in God was crucial in assisting nurses to overcome numerous obstacles and offered them peace of mind while they cared for people with COVID-19 [78]. This emphasizes the importance of a spiritual therapeutic environment in the workplace in overcoming pandemic obstacles and accomplishing duties. However, this contradicts an earlier study that found a reduced level of faith in God among crisis-affected individuals [79], perhaps due to inadequate consideration of faith in comparison to physical and psychological aspects.

Nurses should be provided with social and organizational support since it diminishes their emotional exhaustion and fears [80]. Family support is also essential for nurses to cope with psychological challenges during epidemic outbreaks; psychological support can positively affect nurses’ feelings and emotions; support from colleagues, friends, and families provides nurses with the opportunity to avoid negative emotions and feelings and diminishes the danger of burnout; and social support strengthens resilience and decreases feelings of isolation [81]. Socially and organizationally supported nurses expect to receive adequate and clear knowledge about the pandemic [82]. Huerta-González et al. (2021) provided the following recommendations: it is necessary to regularly monitor the psychological well-being of frontline nurses and use psychological counselling and interventions to prevent the adverse psychological experience of nurses [83]. Nurses should pay attention to their need for drinks, food, and breaks. While at work, nurses should step away for short breaks from time to time. They should use calming strategies. Meditation techniques are also helpful. Nurses should pay attention to the psychological well-being of new team members. In addition, they should provide support conversations to their colleagues. Nurses should search for opportunities to boost the well-being of other team members and help new team members feel safe and valued.

It makes sense to ask managers to create a comfortable staff break room. It is advisable to have regular meetings to address current issues related to the psychological well-being of team members. Managers and leaders of organizations should be approachable and invite feedback from the team. They should regularly communicate with nurses. If necessary, managers should use the help of professional psychologists to provide psychological support to their subordinates. Managers should systematically monitor the psychological health of staff and rotate nurses between high- and low-stress functions. Managers should support flexible work schedules and shorter working shifts. Managers should ensure that nurses have enough time to rest. Managers must ensure that the experience of more experienced colleagues is transferred to less experienced ones.

Additionally, it is necessary to provide nurses with training programs and help nurses to be prepared for the challenges of future pandemics; nurses should be updated with the latest information about the pandemic [84]. Al Maqbali et al. (2021) recommend providing nurses with training material and online workshops to enable them to cope with any psychological problems [26]. In addition, organizations should improve working conditions for nurses through resource allocation, implementing flexible schedules, rotating nurses, and using effective communication. Nurses should be provided with an adequate education. The content of the training should include the use of PPE, ward disinfection, hand hygiene, medical sterilization, and waste management [13]. It is reasonable to establish an infection control system and monitor in real time the behavior of nurses in terms of infection prevention and the use of PPE [13]. There is still a need for health organizations to be cognizant of the signals that nurses may be giving post their COVID-19 experience, even though the pandemic itself may be over. A focused discussion with nurses about their stress management strategies is a powerful tool for them.

## 5. Limitations

Despite the study’s insightful findings, the inherent limitation of qualitative studies is that their small samples render the results not generalizable [85]. The number of available beds, the patient population, and the cultural norms of the area all play a role in shaping a nurse’s daily routine and make her experiences at one hospital vastly different from those at another. Furthermore, questions were asked about the participants’ recollections of events as far back as a year before the data collection. As some time had passed since the study began, it is possible that participants’ memories were less reliable than they once had been. In addition, the crisis of COVID-19 is a phenomenological one, unique to the period and place in which it occurred. Extrapolating these results to incorporate future calamities requires extreme caution.

## 6. Conclusions

Overall, this study provides insights into the impact of witnessing patients’ death during stressful times on female nurses. This experience refined a number of nurses’ skills and enhanced a few abilities. They have become more adaptable and capable of functioning in various teams comprised of individuals with varying skill levels. They demonstrated growing in their professional identity, gratitude, and spirituality. However, lack of knowledge about pandemics, effective adaptive mechanisms, and social support resources exacerbated psychological symptoms among participants. It is necessary to provide nurses with training programs and help them to be prepared for the challenges of future pandemics. Emotional fatigue can be reduced by providing nurses with social and organizational support through regular meetings to discuss current concerns affecting their mental health.

## Figures and Tables

**Table 1:** Emerged themes and subthemes (*n* = 7).

	Themes	Subthemes
Theme 1	Witnessing Sudden Death of the Patient	Emotional and Psychological Trauma (*n* = 6)
		Helplessness and Uncertainty (*n* = 6)
		Guilt and Self-Reproach (*n* = 7)
Theme 2	Aftershock Reactions	Somatization as Defence Mechanism (*n* = 4)
		Withdrawal/Isolation (*n* = 4)
		Emotional Disengagement (*n* = 7)
		Self-Denial (5)
Theme 3	Challenges and Obstacles	Struggles in Communication (*n* = 3)
		Compromised Protocols (*n* = 4)
		Blind Shooting (*n* = 4)
		Attachment to the Patient and Family (*n* = 6)
Theme 4	Victorious and Celebrate Life	(*n* = 7)
Theme 5	Calm After the Storm	Strengthened Faith in God (*n* = 5)
		Valuing Life and Family (*n* = 5)
		Compassionate Empathy (*n* = 6)
		Game Changer (*n* = 6)
Theme 6	Emerging Aptitude	Transformative Competence (*n* = 7)
		Self-Effacing Beyond the Call of Duty (*n* = 6)
		Stoical Engagement (*n* = 4)

## Data Availability

The data that support the findings of this study are available from the corresponding author upon reasonable request.

## References

[1] AlAteeq D.A., Aljhani S., Althiyabi I., Majzoub S. (2020). Mental health among healthcare providers during coronavirus disease (COVID-19) outbreak in Saudi Arabia. J. Infect. Public Health.

[2] Mohammed A., Sheikh T.L., Poggensee G., Nguku P., Olayinka A., Ohuabunwo C., Eaton J. (2015). Mental health in emergency response: Lessons from Ebola. Lancet Psychiatry.

[3] Hsieh K.-Y., Kao W.-T., Li D.-J., Lu W.-C., Tsai K.-Y., Chen W.-J., Chou L.-S., Huang J.-J., Hsu S.-T., Chou F.H.-C. (2020). Mental health in biological disasters: From SARS to COVID-19. Int. J. Soc. Psychiatry.

[4] Xiang Y.-T., Yang Y., Li W., Zhang L., Zhang Q., Cheung T., Ng C.H. (2020). Timely mental health care for the 2019 novel coronavirus outbreak is urgently needed. Lancet Psychiatry.

[5] Petzold M.B., Bendau A., Plag J., Pyrkosch L., Maricic L.M., Betzler F., Rogoll J., Große J., Ströhle A. (2020). Risk, resilience, psychological distress, and anxiety at the beginning of the COVID-19 pandemic in Germany. Brain Behav..

[6] Kackin O., Ciydem E., Aci O.S., Kutlu F.Y. (2020). Experiences and psychosocial problems of nurses caring for patients diagnosed with COVID-19 in Turkey: A qualitative study. Int. J. Soc. Psychiatry.

[7] Jaiswal A., Singh T., Arya Y.K. (2020). “Psychological Antibodies” to Safeguard Frontline Healthcare Warriors Mental Health Against COVID-19 Pandemic-Related Psychopathology. Front. Psychiatry.

[8] Vindegaard N., Benros M.E. (2020). COVID-19 pandemic and mental health consequences: Systematic review of the current evidence. Brain Behav. Immun..

[9] Portoghese I., Galletta M., Meloni F., Piras I., Finco G., D’Aloja E., Campagna M. (2021). Dealing With COVID-19 Patients: A Moderated Mediation Model of Exposure to Patients’ Death and Mental Health of Italian Health Care Workers. Front. Psychol..

[10] Jackson D., Bradbury-Jones C., Baptiste D., Gelling L., Morin K., Neville S., Smith G.D. (2020). Life in the pandemic: Some reflections on nursing in the context of COVID-19. J. Clin. Nurs..

[11] Maben J., Bridges J. (2020). Covid-19: Supporting nurses’ psychological and mental health. J. Clin. Nurs..

[12] Smith G.D., Ng F., Li W.H.C. (2020). COVID-19: Emerging compassion, courage and resilience in the face of misinformation and adversity. J. Clin. Nurs..

[13] Huang L., Lin G., Tang L., Yu L., Zhou Z. (2020). Special attention to nurses’ protection during the COVID-19 epidemic. Crit. Care.

[14] Rathnayake S., Dasanayake D., Maithreepala S.D., Ekanayake R., Basnayake P.L. (2021). Nurses’ perspectives of taking care of patients with Coronavirus disease 2019: A phenomenological study. PLoS ONE.

[15] Kisely S., Warren N., McMahon L., Dalais C., Henry I., Siskind D. (2020). Occurrence, prevention, and management of the psychological effects of emerging virus outbreaks on healthcare workers: Rapid review and meta-analysis. BMJ.

[16] Gunawan Peristiowati Y., Ellina A.D. (2021). Stress of Nurses during the COVID-19 Pandemic: A Literature Review. Int. J. Sci. Soc..

[17] Bagheri Sheykhangafshe F., Saeedi M., Ansarifar N., Savabi Niri V., Deldari Alamdari M. (2021). Evaluation of post-traumatic stress disorder, depression and anxiety of nurses during coronavirus 2019 pandemic: A systematic review. Iran. J. Nurs. Res..

[18] Mahalli S., Nematifard T. (2021). Mental Health Status of Nurses During the COVID-19 Pandemic: A Systematic Review. Ment. Health.

[19] Ardebili M.E., Naserbakht M., Bernstein C., Alazmani-Noodeh F., Hakimi H., Ranjbar H. (2020). Healthcare providers experience of working during the COVID-19 pandemic: A qualitative study. Am. J. Infect. Control.

[20] Arnetz J.E., Goetz C.M., Arnetz B.B., Arble E. (2020). Nurse Reports of Stressful Situations during the COVID-19 Pandemic: Qualitative Analysis of Survey Responses. Int. J. Environ. Res. Public Health.

[21] Liu S., Liu Y., Liu Y. (2020). Somatic symptoms and concern regarding COVID-19 among Chinese college and primary school students: A cross-sectional survey. Psychiatry Res..

[22] Tan R., Yu T., Luo K., Teng F., Liu Y., Luo J., Hu D. (2020). Experiences of clinical first-line nurses treating patients with COVID-19: A qualitative study. J. Nurs. Manag..

[23] Alnazly E., Khraisat O.M., Al-Bashaireh A.M., Bryant C.L. (2021). Anxiety, depression, stress, fear and social support during COVID-19 pandemic among Jordanian healthcare workers. PLoS ONE.

[24] Wilson W., Raj J.P., Rao S., Ghiya M., Nedungalaparambil N.M., Mundra H., Mathew R. (2020). Prevalence and Predictors of Stress, anxiety, and Depression among Healthcare Workers Managing COVID-19 Pandemic in India: A Nationwide Observational Study. Indian J. Psychol. Med..

[25] Chowdhury S.R., Sunna T.C., Das D.C., Kabir H., Hossain A., Mahmud S., Ahmed S. (2021). Mental health symptoms among the nurses of Bangladesh during the COVID-19 pandemic. Middle East Curr. Psychiatry.

[26] Al Maqbali M., Al Sinani M., Al-Lenjawi B. (2020). Prevalence of stress, depression, anxiety and sleep disturbance among nurses during the COVID-19 pandemic: A systematic review and meta-analysis. J. Psychosom. Res..

[27] Nowrouzi-Kia B., Sithamparanathan G., Nadesar N., Gohar B., Ott M. (2021). Factors associated with work performance and mental health of healthcare workers during pandemics: A systematic review and meta-analysis. J. Public Health.

[28] Colaizzi P.F., Valle R.S., King M. (1978). Psychological research as the phenomenologist views it. Existential-Phenomenological Alternatives for Psychology.

[29] Ellickson-Larew S.A., Carney J.R., Coady A.T., Barnes J.B., Grunthal B., Litz B.T. (2022). Trauma-And Stressor-Related Disorders.

[30] Snoek A., McGeer V., Brandenburg D., Kennett J. (2021). Managing shame and guilt in addiction: A pathway to recovery. Addict Behav..

[31] Adams H.E., Sutker P.B. (2007). Comprehensive Handbook of Psychopathology.

[32] Van der Wee N.J., Bilderbeck A.C., Cabello M., Ayuso-Mateos J.L., Saris I.M., Giltay E.J., Penninx B.W., Arango C., Post A., Porcelli S. (2019). Working definitions, subjective and objective assessments and experimental paradigms in a study exploring social withdrawal in schizophrenia and Alzheimer’s disease. Neurosci. Biobehav. Rev..

[33] Javed S., Parveen H. (2021). Adaptive coping strategies used by people during coronavirus. J. Educ. Health Promot..

[34] Xu H., Stjernswärd S., Glasdam S. (2021). Psychosocial experiences of frontline nurses working in hospital-based settings during the COVID-19 pandemic—A qualitative systematic review. Int. J. Nurs. Stud. Adv..

[35] Abdurrahman M., Hrobsky H. (2020). Recommendations and Resources for Coping with Burnout. Humanism and Resilience in Residency Training.

[36] Manela T. (2016). Negative feelings of gratitude. J. Value Inq..

[37] Bandura A., Freeman W.H., Lightsey R. (1999). Self-Efficacy: The Exercise of Control. J. Cogn. Psychother..

[38] MacLellan A., Brown M.E.L., LeBon T., Guha N. (2022). The Application of Stoicism to Health Professions Education. Applied Philosophy for Health Professions Education.

[39] Fan J., Hu K., Li X., Jiang Y., Zhou X., Gou X., Li X. (2020). A qualitative study of the vocational and psychological perceptions and issues of transdisciplinary nurses during the COVID-19 outbreak. Aging.

[40] Liu Q., Luo D., Haase J.E., Guo Q., Wang X.Q., Liu S., Xia L., Liu Z., Yang J., Yang B.X. (2020). The experiences of health-care providers during the COVID-19 crisis in China: A qualitative study. Lancet Glob. Health.

[41] Okediran J.O., Ilesanmi O.S., Fetuga A.A., Onoh I., Afolabi A.A., Ogunbode O., Olajide L., Kwaghe A.V., Balogun M.S. (2020). The experiences of healthcare work-ers during the COVID-19 crisis in Lagos, Nigeria: A qualitative study. Germs.

[42] Sheng Q., Zhang X., Wang X., Cai C. (2020). The influence of experiences of involvement in the COVID-19 rescue task on the professional identity among Chinese nurses: A qualitative study. J. Nurs. Manag..

[43] Kellogg M.B., Scherr A.E.S., Ayotte B.J. (2021). “All of this was awful”: Exploring the experience of nurses caring for patients with COVID-19 in the United States. Nurs. Forum.

[44] Copel L.C., Lengetti E., McKeever A., Pariseault C.A., Smeltzer S.C. (2022). An uncertain time: Clinical nurses’ first impressions during the COVID-19 pandemic. Res. Nurs. Health.

[45] Sun N., Wei L., Shi S., Jiao D., Song R., Ma L., Wang H., Wang C., Wang Z., You Y. (2020). A qualitative study on the psychological experience of caregivers of COVID-19 patients. Am. J. Infect. Control.

[46] Muz G., RN G.E.Y., Rn G.M. (2021). Experiences of nurses caring for patients with COVID-19 in Turkey: A phenomenological enquiry. J. Nurs. Manag..

[47] Gao X., Jiang L., Hu Y., Li L., Hou L. (2020). Nurses’ experiences regarding shift patterns in isolation wards during the COVID-19 pandemic in China: A qualitative study. J. Clin. Nurs..

[48] Şimşek D.C., Günay U. (2021). Experiences of nurses who have children when caring for COVID-19 patients. Int. Nurs. Rev..

[49] Lee N., Lee H.-J. (2020). South Korean Nurses’ Experiences with Patient Care at a COVID-19-Designated Hospital: Growth after the Frontline Battle against an Infectious Disease Pandemic. Int. J. Environ. Res. Public Health.

[50] Deliktas Demirci A., Oruc M., Kabukcuoglu K. (2020). ‘It was difficult, but our struggle to touch lives gave us strength’: The experience of nurses working on COVID-19 wards. J. Clin. Nurs..

[51] Bennett P., Noble S., Johnston S., Jones D., Hunter R. (2020). COVID-19 confessions: A qualitative exploration of healthcare workers experiences of working with COVID-19. BMJ Open.

[52] Goh Y., Yong Q.Y.J.O., Chen T.H., Ho S.H.C., Chee Y.I.C., Chee T.T. (2020). The Impact of COVID-19 on nurses working in a University Health System in Singapore: A qualitative descriptive study. Int. J. Ment. Health Nurs..

[53] Sethi A., Aamir H.S., Sethi B.A., Ghani N., Saboor S. (2020). Impact on Frontline Nurses in the Fight against Coronavirus Disease. Ann. King Edw. Med. Univ..

[54] Vindrola-Padros C., Andrews L., Dowrick A., Djellouli N., Fillmore H., Gonzalez E.B., Javadi D., Lewis-Jackson S., Manby L., Mitchinson L. (2020). Perceptions and experiences of healthcare workers during the COVID-19 pandemic in the UK. BMJ Open.

[55] Duarte J., Pinto-Gouveia J. (2017). Empathy and feelings of guilt experienced by nurses: A cross-sectional study of their role in burnout and compassion fatigue symptoms. Appl. Nurs. Res..

[56] Hofmeyer A., Taylor R., Kennedy K. (2020). Knowledge for nurses to better care for themselves so they can better care for others during the Covid-19 pandemic and beyond. Nurse Educ. Today.

[57] Galanti C. (2022). National heroes, disposable workers. How collective action in the health and social care sector during the pandemic negotiated with the self-sacrificing worker ideal. Gend. Work. Organ..

[58] Kim Y. (2018). Nurses’ experiences of care for patients with Middle East respiratory syndrome-coronavirus in South Korea. Am. J. Infect. Control.

[59] Labrague L.J., de los Santos J. (2020). COVID-19 anxiety among frontline nurses: Predictive role of organizational support, personal resilience and social support. J. Nurs. Manag..

[60] Cardoso M.F.P.T., Martins M.M.F.P.D.S., Trindade L.D.L., Ribeiro O.M.P.L., Fonseca E.F. (2021). The COVID-19 pandemic and nurses’ attitudes toward death. Rev. Lat.-Am. Enferm..

[61] Annaloro C., Arrigoni C., Ghizzardi G., Dellafiore F., Magon A., Maga G., Nania T., Pittella F., Villa G., Caruso R. (2021). Burnout and post-traumatic stress disorder in frontline nurses during the COVID-19 pandemic: A systematic literature review and meta-analysis of studies published in 2020. Acta Biomed.

[62] Wati S.G., Sutrimo A. (2022). Factors associated with burnout among nurses during covid-19 pandemic: A scoping review. J. Keperawatan Respati Yogyak..

[63] Conolly A., Abrams R., Rowland E., Harris R., Couper K., Kelly D., Kent B., Maben J. (2022). “What Is the Matter With Me?” or a “Badge of Honor”: Nurses’ Constructions of Resilience During Covid-19. Glob. Qual. Nurs. Res..

[64] Windle G. (2011). What is resilience? A review and concept analysis. Rev. Clin. Gerontol..

[65] Kirk K., Cohen L., Edgley A., Timmons S. (2021). “I don’t have any emotions”: An ethnography of emotional labour and feeling rules in the emergency department. J. Adv. Nurs..

[66] Li Z., Ge J., Yang M., Feng J., Qiao M., Jiang R., Bi J., Zhan G., Xu X., Wang L. (2020). Vicarious traumatization in the general public, members, and non-members of medical teams aiding in COVID-19 control. Brain Behav. Immun..

[67] Chew N.W.S., Lee G.K.H., Tan B.Y.Q., Jing M., Goh Y., Ngiam N.J.H., Yeo L.L.L., Ahmad A., Ahmed Khan F., Napolean Shanmugam G.N. (2020). A multinational, multicentre study on the psychological outcomes and associated physical symptoms amongst healthcare workers during COVID-19 outbreak. Brain Behav. Immun..

[68] Lou N.M., Montreuil T., Feldman L.S., Fried G.M., Lavoie-Tremblay M., Bhanji F., Kennedy H., Kaneva P., Harley J.M. (2022). Nurses’ and physicians’ distress, burnout, and coping strategies during COVID-19: Stress and impact on perceived performance and intentions to quit. J. Contin. Educ. Health Prof..

[69] Holroyd E., Long N.J., Appleton N.S., Davies S.G., Deckert A., Fehoko E., Laws M., Martin-Anatias N., Simpson N., Sterling R. (2022). Community healthcare workers’ experiences during and after COVID-19 lockdown: A qualitative study from Aotearoa New Zealand. Health Soc. Care Community.

[70] Bergman L., Falk A., Wolf A., Larsson I., Rn C.L.B., Rn C.A.W. (2021). Registered nurses’ experiences of working in the intensive care unit during the COVID-19 pandemic. Nurs. Crit. Care.

[71] Liljestrand R., Martin S. (2021). Stress and Resilience Among Healthcare Workers During the COVID-19 Pandemic. Rehabil. Nurs..

[72] Sun W., Chen W.-T., Zhang Q., Ma S., Huang F., Zhang L., Lu H. (2021). Post-Traumatic Growth Experiences among COVID-19 Confirmed Cases in China: A Qualitative Study. Clin. Nurs. Res..

[73] Victor C.G.P., Treschuk J.V. (2019). Critical Literature Review on the Definition Clarity of the Concept of Faith, Religion, and Spirituality. J. Holist. Nurs..

[74] Pirutinsky S., Cherniak A.D., Rosmarin D.H. (2020). COVID-19, Mental Health, and Religious Coping Among American Orthodox Jews. J. Relig. Health.

[75] Neto G.L., Rodrigues L., da Silva D.A.R., Turato E.R., Campos C.J.G. (2018). Spirituality review on mental health and psychiatric nursing. Rev. Bras. Enferm..

[76] Baksi A., Sürücü H.A. (2019). Is spirituality an important variable as the predictor of emotional labour for nursing students?. Nurse Educ. Today.

[77] Barber C. (2019). Working within a spiritual framework. Br. J. Nurs..

[78] Alquwez N., Cruz J.P., Balay-Odao E.M., Alquwez R.N. (2021). Nurses’ spiritual well-being and the COVID-19 pandemic: A thematic approach. J. Nurs. Manag..

[79] Coppola I., Rania N., Parisi R., Lagomarsino F. (2021). Spiritual Well-Being and Mental Health During the COVID-19 Pandemic in Italy. Front. Psychiatry.

[80] Sirois F.M., Owens J. (2021). Factors Associated With Psychological Distress in Health-Care Workers During an Infectious Disease Outbreak: A Rapid Systematic Review of the Evidence. Front. Psychiatry.

[81] Galanis P., Vraka I., Fragkou D., Bilali A., Kaitelidou D. (2021). Nurses’ burnout and associated risk factors during the COVID-19 pandemic: A systematic review and meta-analysis. J. Adv. Nurs..

[82] Fernandez R., Lord H., Halcomb E., Moxham L., Middleton R., Alananzeh I., Ellwood L. (2020). Implications for COVID-19: A systematic review of nurses’ experiences of working in acute care hospital settings during a respiratory pandemic. Int. J. Nurs. Stud..

[83] Huerta-González S., Selva-Medrano D., López-Espuela F., Caro-Alonso P., Novo A., Rodríguez-Martín B. (2021). The Psychological Impact of COVID-19 on Front Line Nurses: A Synthesis of Qualitative Evidence. Int. J. Environ. Res. Public Health.

[84] Al Thobaity A., Alshammari F. (2020). Nurses on the Frontline against the COVID-19 Pandemic: An Integrative Review. Dubai Med. J..

[85] USC Libraries: Organizing Your Social Sciences Research Paper. https://libguides.usc.edu/writingguide/qualitative.

